# Investigating the Group-Level Impact of Advanced Dual-Echo fMRI Combinations

**DOI:** 10.3389/fnins.2016.00571

**Published:** 2016-12-12

**Authors:** Ádám Kettinger, Christopher Hill, Zoltán Vidnyánszky, Christian Windischberger, Zoltán Nagy

**Affiliations:** ^1^Department of Nuclear Techniques, Budapest University of Technology and EconomicsBudapest, Hungary; ^2^Brain Imaging Centre, Research Centre for Natural Sciences, Hungarian Academy of SciencesBudapest, Hungary; ^3^Laboratory for Social and Neural Systems Research, University of ZurichZurich, Switzerland; ^4^Center for Medical Physics and Biomedical Engineering, Medical University of ViennaVienna, Austria

**Keywords:** fMRI, multi-echo, random-effects analysis, signal dropout, EPI, inter-subject variance

## Abstract

Multi-echo fMRI data acquisition has been widely investigated and suggested to optimize sensitivity for detecting the BOLD signal. Several methods have also been proposed for the combination of data with different echo times. The aim of the present study was to investigate whether these advanced echo combination methods provide advantages over the simple averaging of echoes when state-of-the-art group-level random-effect analyses are performed. Both resting-state and task-based dual-echo fMRI data were collected from 27 healthy adult individuals (14 male, mean age = 25.75 years) using standard echo-planar acquisition methods at 3T. Both resting-state and task-based data were subjected to a standard image pre-processing pipeline. Subsequently the two echoes were combined as a weighted average, using four different strategies for calculating the weights: (1) simple arithmetic averaging, (2) BOLD sensitivity weighting, (3) temporal-signal-to-noise ratio weighting and (4) temporal BOLD sensitivity weighting. Our results clearly show that the simple averaging of data with the different echoes is sufficient. Advanced echo combination methods may provide advantages on a single-subject level but when considering random-effects group level statistics they provide no benefit regarding sensitivity (i.e., group-level *t*-values) compared to the simple echo-averaging approach. One possible reason for the lack of clear advantages may be that apart from increasing the average BOLD sensitivity at the single-subject level, the advanced weighted averaging methods also inflate the inter-subject variance. As the echo combination methods provide very similar results, the recommendation is to choose between them depending on the availability of time for collecting additional resting-state data or whether subject-level or group-level analyses are planned.

## Introduction

Increases in blood oxygenation level dependent (BOLD) signal in functional magnetic resonance imaging (fMRI) are only transient and thus it is advantageous to acquire fMRI data with high temporal resolution. Most often echo planar imaging (EPI) (Mansfield, 1977; Stehling et al., 1991; Ordidge, 1999) is used for data acquisition, and accordingly, fMRI experiments must contend with all the shortcomings of EPI methods (Fischer and Ladebeck, 1998; Turner and Ordidge, 2000). In particular, susceptibility-induced magnetic field gradients within the head can lead to a signal dropout, hence eliminating the possibility of observing the BOLD signal in these areas (Ojemann et al., 1997). A number of approaches have been proposed to recover this lost signal. For example, prescribing oblique slices (Deichmann et al., 2003), employing z-shimming (Ordidge et al., 1994; Weiskopf et al., 2006) or acquiring images at higher resolution (Weiskopf et al., 2007) avoid the signal drop-out depending on whether TE is shifted in the phase or frequency encoding direction by the local susceptibility gradient. These methods however are not necessarily advantageous in all voxels containing brain tissue and may even be disadvantageous for voxels that are not in the vicinity of susceptibility induced magnetic field gradients.

Multi-echo fMRI acquisitions propose to sample the BOLD response in all voxels in an image volume by acquiring several EPIs with varying echo times (TEs) after each excitation (Posse et al., 1999). The final time-series is formed as a weighted average of data obtained with the different TEs where the weights are specific for each voxel to provide the highest sensitivity for detecting the BOLD signal (Posse et al., 1999; Poser et al., 2006). As few as two images with different TEs have been used previously (Glover and Law, 2001; Schwarzbauer et al., 2010).

Despite this convincing argument, the actual demonstration in multi-subject experiments, targeting average group effects is still lacking. Surprisingly, a recent study (Kirilina et al., 2016) reported that several advanced fMRI acquisition methods failed to provide the expected advantages in random effects group-level analyses (Friston et al., 2007). They contrasted the standard 2D single echo fMRI acquisition method against 3D and multi-echo variants. Even though both 3D and multi-echo acquisition methods had been shown to provide advantages in experiments involving individual subjects, the group-level results indicated that multi-echo acquisition methods only provided an advantage in areas with susceptibility-related signal drop out (e.g., the orbitofrontal cortex) (Kirilina et al., 2016). Hence, the aims of the present paper are twofold:

1. Ascertain that optimal combination of dual-echo fMRI data increases BOLD sensitivity on an individual level.
2. Investigate whether the so-achieved optimal BOLD sensitivity carries over to random-effects group-level analyses.

Herein we address these two aims by assessing fMRI activation maps on the single-subject and group level using various combinations of dual-echo data sets obtained at 3 Tesla.

## Materials and methods

### Volunteers

The Kantonale Ethics Komitee (i.e., regional ethics committee) of Zurich approved involvement of the human volunteers and each participant signed a written informed consent before the experiment.

Thirty (mean age = 24.8 years, std = 1.8 years, 15 male) right-handed, non-smoking, medication-free volunteers participated in the study. Each had normal eyesight and no history of neurological disorders. The MRI or the physiological data from three participants were compromised due to computer disk error or physiological sensor failure. Because the physiological data were used for correcting time-series artifacts, henceforth this paper pertains to the remaining 27 participants (14 male, mean age = 25.75 years, std = 1.77 years).

### Data acquisition

All MRI data were acquired on a 3T Philips Achieva scanner (Philips Healthcare, Best, The Netherlands) equipped with an 8-channel receive-only head coil and single-channel body transmit coil. For both the resting-state and the task-based fMRI data sets 36 axial slices were collected at 2.6 mm thickness using single-shot double-echo GE-EPI with twofold SENSE acceleration (Pruessmann et al., 1999) in the phase encoding direction and TE1/TE2 = 17/44 ms, TR = 2.6 s, FOV 200 × 200 mm^2^, in-plane resolution 2.5 × 2.5 mm^2^, slice gap 0.6 mm. The total EPI readout duration for each echo was 24.792 ms. For each subject the resting-state data set contained 116 image volumes while the task-based fMRI data were acquired twice, each set containing 282 image volumes. Cardiac and respiratory signals were concurrently acquired via electrocardiogram and a breathing belt, respectively, to allow for removal of fluctuations caused by cardiac pulsation and breathing from the fMRI time-series as part of the image pre-processing pipeline.

During the task-based experiment subjects in the scanner played a competitive game against a human opponent outside of the scanner. Each trial consisted of a jittered choice and feedback epoch, in which the subject in the scanner could either win money or not depending on his/her choice and the choice of the opponent outside of the scanner. This quantity is the parametric modulator of interest, denoted as “reward.” Reward in this game is contingent on the player's ability to predict his opponent's behavior on any given round in order to be rewarded. Scanned participants played 160 trials of the inspection game in two sessions of 80 trials paired with another player seated in an adjacent room. Each trial began with a fixation-cross presented for 1.25 to 8 s, followed by a decision screen featuring two pictograms describing choice options. Participants had up to 2 s to make their decision and played simultaneously. Decisions were confirmed with a red square, which was displayed for a minimum of 100 ms, and up to the time it took for both answers to be recorded. After each player made his or her decision, a fixation cross was presented for 1.25 to 8 s, following which the feedback screen was displayed for 2.5 s. The average duration of a trial summed up to 9 s. fMRI contrast sensitivity was optimized by simulating optimal task ITIs. In total, the task lasted 25 min. It is noteworthy that the “reward”-based analysis elicits robust BOLD responses in dopamergetic pathways (Nucleus Accumbens, Substantia Nigra, Ventral Tegmental Area) and orbitofrontal cortex. We direct the interested reader toward Neurosynth (Yarkoni et al., 2011) for a meta-analytic overview for the term “Reward” across 671 studies as of October 24, 2016.

### fMRI regressors

The fMRI dataset used for comparing echo combination procedures was modeled with regressors capturing latent variables from a computational model described previously (Hampton et al., 2008). Briefly, *Expected Value of the Chosen option* refers to the subject's prediction about his/her probability of winning the given his choice. *First-order prediction error* refers to the subject's estimate of observing a certain action from the opponent, given the opponent's history of choices. *Influence update* refers to the estimated effect of the player's choice on the opponent's model of the player.

### SPM design matrix

Our design matrix included the onset of the decision period with *expected value of the chosen options* as parametric modulator and the onset of the feedback epoch with reward, *first-order prediction error*, and *influence update* as parametric modulators. Orthogonalization was turned off in order to capture the variance uniquely explained by each factor. In addition, we included, as nuisance regressors, all six movement parameters and physiological fluctuations related to heart rate, and breathing with the procedure described below. We constructed a boxcar epoch function whose duration corresponds to the reaction time of the decision to optimally account for variability in decision time. The feedback epoch was modeled using stick functions.

### Pre-processing of fMRI time-series data

For pre-processing and statistical analysis SPM12 (Wellcome Trust Centre for Neuroimaging, UCL, UK), and Matlab (The MathWorks, MA, USA) were used.

First, physiological time-series were transformed following the RETROICOR procedure (Glover et al., 2000), as implemented in TAPAS, an open source software package (http://www.translationalneuromodeling.org/tapas) that uses Fourier expansions of various orders for the phases of cardiac pulsation (3rd order), respiration (4th order), and cardio-respiratory interaction (1st order) (Harvey et al., 2008). A cardio-respiratory interaction refers to the respiratory time-series multiplied by the cardiac time-series to form the interaction term. This is done in order to account for physiological noise not captured by the respective main effects of breathing and heart rate. Subsequently both the resting-state time-series and the two runs of the task-based datasets were realigned with Matlab such that all but the first volumes with short TE were rigid body aligned to the first image and the resulting realignment parameters were used to transform the long TE images identically. Finally, second-order polynomial de-trending was performed right after re-alignment but before calculation of the weights (see “*Echo combinations of resting-state fMRI data*” below).

Additionally, a high-pass filter with a cut-off frequency of 1/128 s was applied to the task-based data after echo combination, co-registration and normalization and immediately before the first-level statistical analysis.

### Generation of gray matter mask

To extract voxel values specific to the gray matter (GM) segment of the brain a binary mask was created by segmenting (Ashburner and Friston, 2005) and normalizing the T_1_-weighted anatomical image of each subject in SPM. Subsequently the GM segment was smoothed by a 6-mm isotropic Gaussian kernel and thresholded to contain only voxels with a GM probability of at least at 0.35. Finally, each individual's GM mask was multiplied together to arrive at a group GM mask. The histograms and scatter plots of **Figures 3–5** were produced after multiplying the *t*-value maps, contrast maps and total variance maps voxel-wise by this final GM mask.

### Echo combinations of resting-state fMRI data

The realigned and de-trended resting-state dual echo dataset of each subject was combined voxel-wise as a normalized weighted average of the two echoes to provide a single time-series, where time is represented by *t*

(1) $$\begin{array}{l} type\text{\_}data\left(t\right)=\frac{w_{1}\cdot S_{1}\left(t\right)+w_{2}\cdot S_{2}\left(t\right)}{w_{1}+w_{2}} \end{array}$$

where *S*_1_(*t*) and *S*_2_(*t*) are the signal amplitude of a given voxel in the same spatial and temporal position acquired with *TE*_1_ and *TE*_2_, respectively, while *w*_1_ and *w*_2_ are the corresponding weights and *type* is one of “*AVE*,” “*BS*,” “*tSNR*,” or “*tBS*” representing the four different methods of echo combinations for calculating the weights (see below). Note, the weights, *w*_1_ and *w*_2_, are calculated separately for each time point in Echo combination method #2 below. For the other three echo combination methods they are identical for all time points in the time series.

Echo combination #1 (*AVE*): The first echo combination was a simple average of the two datasets so that the weights were identical for all voxels at all-time points (i.e., *w*_1_ = *w*_2_ = 1).

Echo combination #2 (*BS*): In the second method the weights were calculated voxel-wise and for each time point (*t*) as

(2a) $$\begin{array}{r} w_{1}\left(t\right)=S_{1}\left(t\right)\cdot TE_{1} \end{array}$$

(2b) $$\begin{array}{r} w_{2}\left(t\right)=S_{2}\left(t\right)\cdot TE_{2} \end{array}$$

where *S*_1_(*t*) and *S*_2_(*t*) are defined as in Equation (1) above. This is commonly considered BOLD sensitivity (*BS*) weighting (Posse et al., 1999; Deichmann et al., 2002).

Echo combination #3 (*tSNR*): The third method used the temporal signal-to-noise ratio (*tSNR*) as weights calculated separately for the two TEs by dividing the temporal mean of a voxel time-series with its temporal standard deviation

(3a) $$\begin{array}{r} w_{1}=tSNR_{1} \end{array}$$

(3b) $$\begin{array}{r} w_{2}=tSNR_{2} \end{array}$$

where *tSNR*_1_ and *tSNR*_2_ are the voxel-wise temporal signal-to-noise values for short (*TE*_1_) and long (*TE*_2_) echo data respectively.

Echo combination #4 (*tBS*): The final scheme merges Echo combinations #2 and #3 as in Poser et al. (2006) and is termed temporal BOLD sensitivity (*tBS*)

(4a) $$\begin{array}{r} w_{1}=tBS_{1}=tSNR_{1}\cdot TE_{1} \end{array}$$

(4b) $$\begin{array}{r} w_{2}=tBS_{2}=tSNR_{2}\cdot TE_{2} \end{array}$$

where *tBS*_1_ and *tBS*_2_ are the temporal-BOLD-contrast-to-noise ratio values, while *tSNR*_1_ and *tSNR*_2_ are as in Echo combination #3 above.

In each of the above echo combination methods the result is a single time-series of resting-state fMRI data. BOLD sensitivity is often defined as in Equation (2) above (Deichmann et al., 2002) and used to quantitatively compare fMRI protocols. Because the datasets have no single effective TE after dual-echo combination (e.g., *AVE_data*), a pseudo measure of the temporal BOLD sensitivity (*ptBS*) was calculated as

(5) $$\begin{array}{l} type\text{\_}ptBS=\frac{mean\left\{\frac{w_{1}\cdot S_{1}\cdot TE_{1}+w_{2}\cdot S_{2}\cdot TE_{1}}{w_{1}+w_{2}}\right\}}{std\left\{\frac{w_{1}\cdot S_{1}+w_{2}\cdot S_{2}}{w_{1}+w_{2}}\right\}} \end{array}$$

where *type* is one of “*AVE*,” “*BS*,” “*tSNR*,” or “*tBS*” to represent one of the four echo combination methods above, *w*_1_ and *w*_2_ are the pair of weights of the corresponding *type* of echo combination method, *S*_1_ and *S*_2_ are voxel signal intensities for the short (*TE*_1_) and long (*TE*_2_), respectively, and both the mean and the standard deviation are calculated across time. Note that in the case *BS*-based echo combination method the *ptBS* measure will depend on the square of the voxel signal and that in this case each time point will have a unique weight.

In order to create group-averaged results the *AVE_ptBS* map was co-registered to the corresponding T_1_-weigthed anatomical image for each subject. Given that each of the four types of *ptBS* maps are calculated from the same data these maps are perfectly aligned. Therefore, the coregistration parameters from *AVE_ptBS* were used for the other three *ptBS* maps. Subsequently the T_1_-weighted anatomical image was normalized in SPM12 to the Montreal Neurological Institute (MNI) template and the normalization parameters were written onto each *ptBS* map. No spatial smoothing was applied to the normalized images.

### Echo combinations of task-based fMRI data

Similarly to the resting-state data, realigned and de-trended task-based time-series with two *TE*s were also combined as a weighted sum using Equation (1). Echo combinations #1 and #2 were performed by using the task-based data for calculating weights, *w*_1_ and *w*_2_, as well as extracting *S*_1_ and *S*_2_.

In Echo combinations #3 and #4 participant-specific resting-state data were used to calculate the weights, *w*_1_ and *w*_2_, but the task-based data were used for extracting *S*_1_ and *S*_2_ for Equation (1). This is because Echo combinations #3 and #4 rely on *tSNR* (Equations 3a,b) for calculating the weights. Hence to avoid including the BOLD response of the task-based time-series into the calculation of the temporal standard deviation, and in turn the weights, the resting-state data were used. More specifically, the *tSNR*_1_ map of *TE*_1_ derived from the resting-state data was realigned with the temporal mean of the realigned task-based data with *TE*_1_. These movement parameters were then applied to the *tSNR*_2_ map to bring it in line with the corresponding task-based data with *TE*_2_.

The four combined task-based datasets were corrected for the different timing of the slice acquisition by temporal interpolation relative to the acquisition time of the slice in the center of the volume using the standard slice time correction method in SPM12 (Sladky et al., 2011).

All four slice-time corrected time-series were normalized to the (MNI) template detailed as follows. The time-mean of the echo averaging combination was co-registered to the T_1_-weighted anatomical image and the estimated parameters were applied to all volumes of all four combined time-series. Normalization to MNI space was performed based on the anatomical image using the standard method in SPM12. All volumes were interpolated to the isotropic resolution of 3 mm during the normalization step and were subsequently smoothed with an isotropic Gaussian kernel with 6 mm FWHM.

### Quantification of resting-state fMRI data

To quantitatively assess the possible advantages of the four different echo combination methods, voxel-wise mean and voxel-wise standard deviation were computed across the subjects for each of the four normalized *ptBS* maps. Voxel-wise ratio of the resulting mean and SD maps were calculated for all combination methods. *AVE_ptBS* was considered the reference to which the other 3 echo combinations were compared. To extract group-level quantitative results, each of the four *ptBS* maps was multiplied by the group GM mask and the mean and standard deviation were calculated from voxels within the mask

### Extracting three regions of interest

The ROIs were generated with http://www.neurosynth.org (Yarkoni et al., 2011) using a default threshold for reverse reference. The first ROI corresponds to the keyword “Reward” (671 studies, 2291 activations). The second ROI we used keywords “Default Mode” (516 studies, 18723 activations). For the third ROI we isolated the orbitofrontal cluster from the first ROI to obtain reward-related orbitofrontal activations. Each of these ROIs was multiplied with the GM mask. The resulting intersection was then applied to the *t*-value difference maps before generation of the histograms.

### Statistical analysis of task-based fMRI data

Single subject general linear model (GLM) analysis was performed separately on each of four pre-processed combined time-series datasets (i.e., *AVE_data, BS_data, tSNR_data, tBS_data*) of each subject. This GLM included, 8 regressors and 24 covariates (physiology and movement) per session. Analysis was performed for one contrast defined as the sum of the two predictors representing reward in the two runs contrasted to baseline.

Random-effects group-level analyses were performed separately for each echo-weighting method using the corresponding single-subject contrast maps.

We calculated voxel-wise *t*-value differences between the simple echo averaging and the other three echo combination methods. Scatter plots of these *t* value difference maps were created from voxels inside the GM mask. Histograms were created both from within the entire GM mask as well as from within the three ROIs of interest.

To further scrutinize the effects of echo combination methods, additional analyses were performed on the *t*-values obtained from random-effects statistical analyses (Friston et al., 2007). By definition these *t*-values are affected by both the mean contrast values as well as the parameter variance. In order to disentangle the two driving factors for the different echo combinations, histograms of voxel-wise differences of contrast and variance maps were also generated for the values of

(6a) $$\begin{array}{r} 100\cdot \frac{contrast_{type}-contrast_{AVE}}{contrast_{type}+contrast_{AVE}}\cdot 2 \end{array}$$

(6b) $$\begin{array}{r} 100\cdot \frac{variance_{type}-variance_{AVE}}{variance_{type}+variance_{AVE}}\cdot 2 \end{array}$$

where *AVE* represents the echo averaging method while *type* represents one of the other three methods for combining the dual-echo data (*BS, tSNR, tBS*). Both the contrast maps and the variance maps were multiplied by the GM mask before histogram calculations.

To estimate and compare the relative contributions of inter-subject vs. intra-subject variance we followed the procedure outlined in Kirilina et al. (2016), which is based on the fact that he total variance of the random effects statistical analysis is expressible as a sum of inter-subject and intra-subject variance components.

Thus far, comparisons of group-level analysis results of statistical analyses were presented. We also performed three separate statistical analyses to compare the advanced echo combination methods with *AVE*. First, first-level analysis was performed for each subject by treating each the four echo-weighting methods as four different sessions. To remove any overall scaling and/or change in the variance of the resulting parameter maps between the combinations, the parameters were voxel-wise divided by their inter-subject standard deviation, separately for each echo combination. Next, from the rescaled first-level parameters three contrast maps were calculated for each subject by taking the difference of the parameters representing reward in one of the advanced echo combination method vs. that of *AVE*. Finally, standard group-level random-effects analysis was performed on these contrast maps separately.

## Results

### Resting-state fMRI data

**Figure 1:**
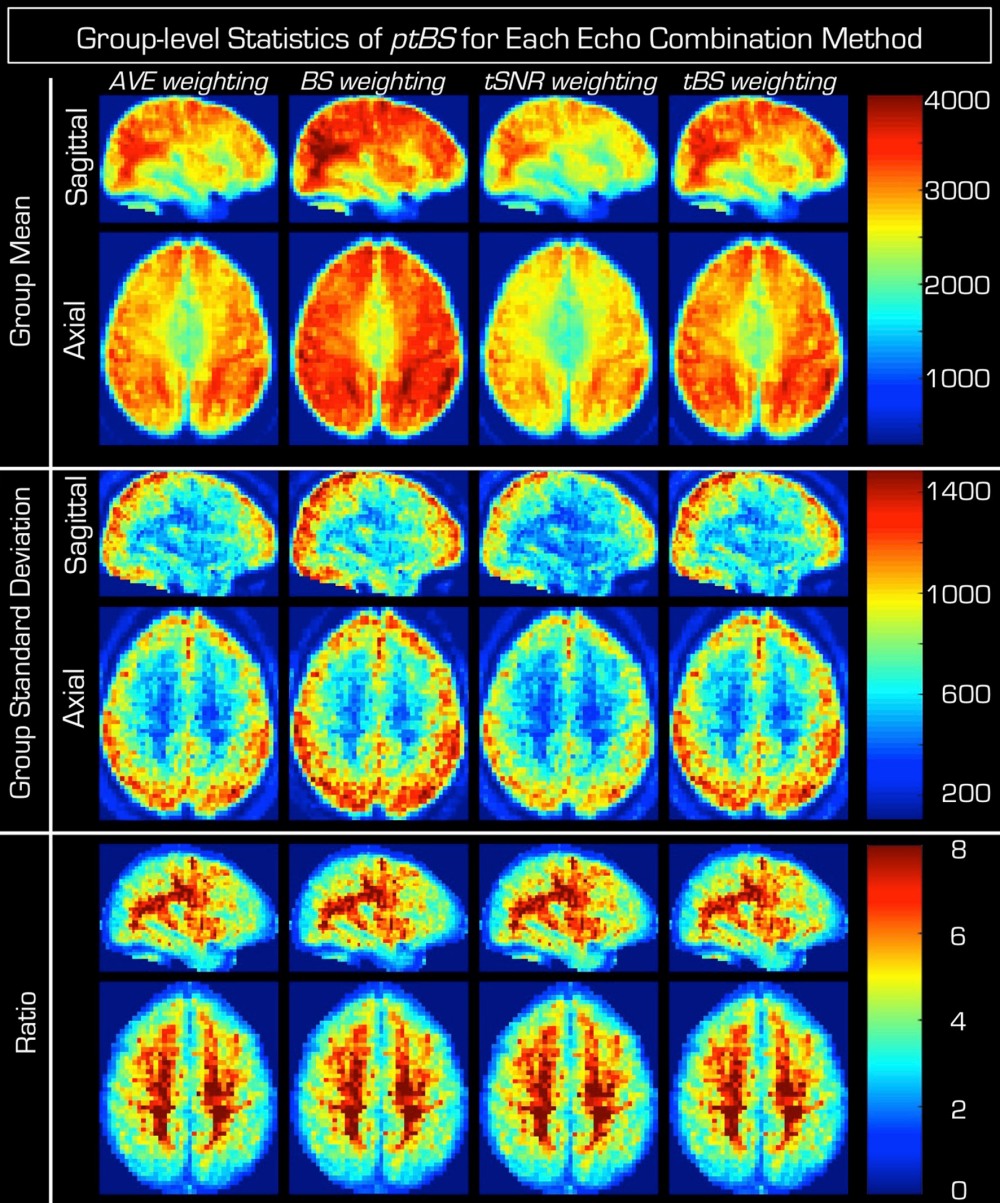
**Group results of the different echo combinations of the resting-state dual echo fMRI data**. The columns represent the four different echo combination methods, where, the pseudo BOLD sensitivity measure (Equation 5) (from left to right *AVE_ptBS, BS_ptBS, tSNR, ptBS*, and *tBS_ptBS*) from each individual in the group is normalized to a common space and subsequently the mean (**top**) and standard deviation (**middle**) across the whole group as well as the ratio of mean and standard deviation (**bottom**) are presented. Note that if for a given echo combination an increase is observed in the average BOLD sensitivity, this increase is accompanied by a similar inflation of the standard deviation across the group (especially so within the gray matter). This is also evident from the ratio images, which are highly similar regardless of the echo combination method used.

According to the top two rows of Figure 1, the advanced echo combination methods have clear benefit on BOLD sensitivity of the resting-state data as assessed by the *type_ptBS* measure (Equation 5) for the advanced echo combination methods *BS* and *tBS* but not for *tSNR*. Out of the four examined echo-weighting strategies the BOLD sensitivity (*BS*) weighting of Echo combination #2 produces the time-series with the highest sensitivity when averaged across the entire group. The *tBS* method of echo combination also produces the expected improvement, which confirms the findings of Poser et al. (2006). For the mean values of the four ptBS maps from within the GM mask please see Table 1.

**Table 1:** **The mean and standard deviation of ptBS values from the resting-state data**.

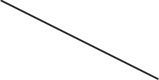	**AVE**	**BS**	**tSNR**	**tBS**
Mean	2537.7	2800.7	2408.5	2637.8
STD	803.7	899.1	733.7	829.7
Ratio	3.2	3.1	3.3	3.2

*STD, standard deviation; Ratio = mean / STD*.

Note however that the center two rows of Figure 1 indicate a similar tendency (especially in the GM of the brain) in the standard deviation of the *ptBS* maps across the subjects, forecasting that a group-level statistical analysis may not benefit from the increased BOLD sensitivity of the advanced echo combination methods (i.e., *BS, tSNR, tBS*). The actual group standard deviation values within the GM mask for the four *ptBS* maps are listed in Table 1. Calculating the voxel-wise ratio supports this hypothesis (bottom two rows of Figure 1).

### Task-based fMRI data

**Figure 2:**
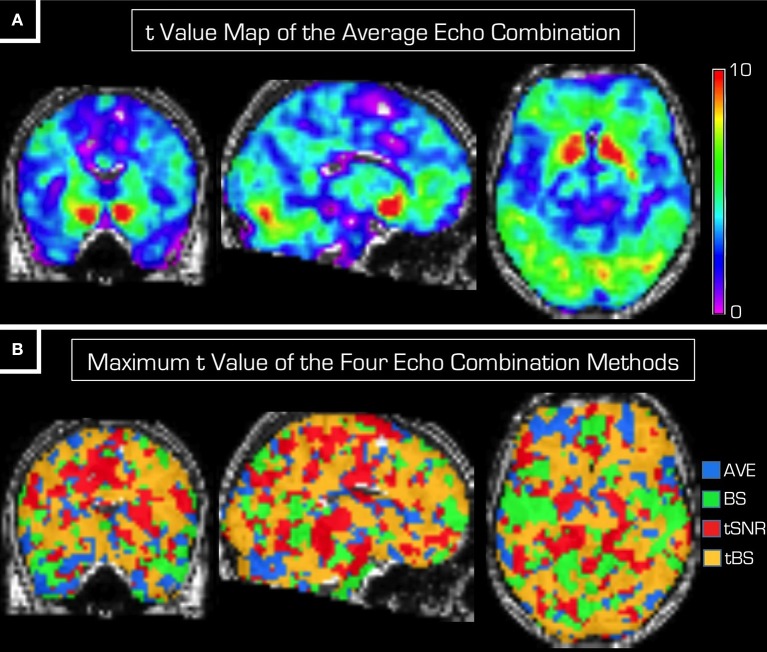
**Random effect analysis results of the task-based fMRI data. (A)** Displays the t map from the statistical analysis based on the *AVE_data* (Equation 1). The robust BOLD response in the ventral striatum is detected as expected. Panel **(B)** is a synthetic image where each voxel is marked by a color (blue = *AVE*, green = *BS*, red = *tSNR* and yellow = *tBS*) that represents the echo combination method that produced the largest *t*-value in that voxel. In about 38% of the voxels within the GM mask, *tBS* echo combination provides the highest *t*-values.

Figure 2A shows the *t*-value map of the group-level random effects-analysis on the time-series data in which the echoes were simply averaged (i.e., *AVE_data*). The group-level results were highly similar when using the time-series data resulting from the other three echo-weighting strategies (data not shown). In Figure 2B the color code in each voxel indicates the echo combination method that provides the largest *t*-value. Within the GM mask the actual percentages were 14.5% for *AVE*, 24.0% for *BS*, 23.3% for *tSNR* and 38.2% *tBS*. Thus, neither of the four echo combination methods comes out as the major winner, not even in the orbitofrontal cortex, where multi-echo data has been shown to provide increased sensitivity (Kirilina et al., 2016).

**Figure 3:**
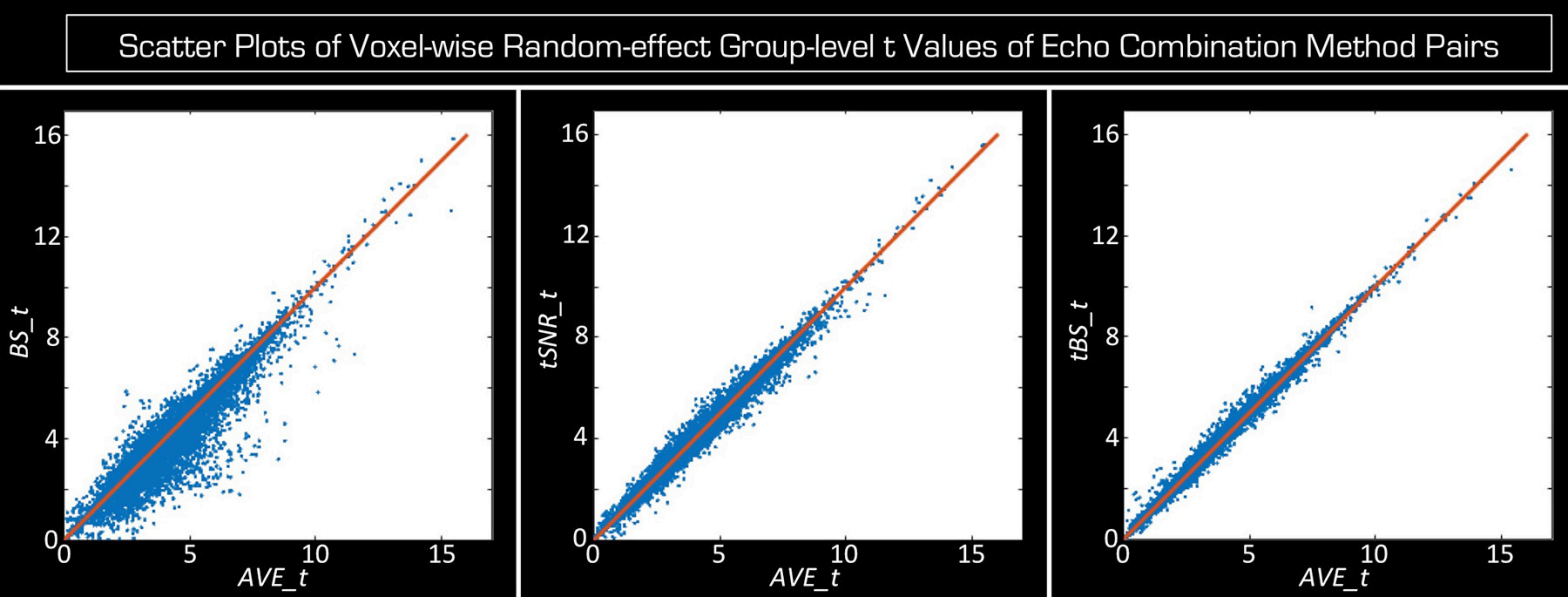
**Scatter plots of the voxel-wise ***t***-values from random effects statistical analyses on data from the four different echo combination methods**. The red line is the identity line. Voxel-wise *t*-values resulting from the analysis based on the *AVE_data* are displayed on the horizontal axis in each subplot. From left to right the vertical axis displays the *t*-values resulting from the statistical analysis based on *BS_data, tSNR_data* and *tBS_data*. Only voxels within the GM mask were considered. Because all points fall near the identity line, neither echo combination method can be declared superior to that of the simple averaging of echoes.

The scatter plots in Figure 3 show the *t*-value of each voxel within the GM mask from the group-level statistical analyses. In each of the three subplots the *t*-value from the average echo combination (i.e., using *AVE_data*) is plotted along the horizontal axis against that resulting from the statistical analysis of each of the other three echo combination methods (*BS_data, tSNR_data* and *tBS_data*) plotted along the vertical axis. Because all points fall in the vicinity of the identity line, neither of the echo combination methods seems superior to simple averaging. It must be noted that the spread around the identity line is broader for the statistical analysis based on *BS_data*.

**Figure 4:**
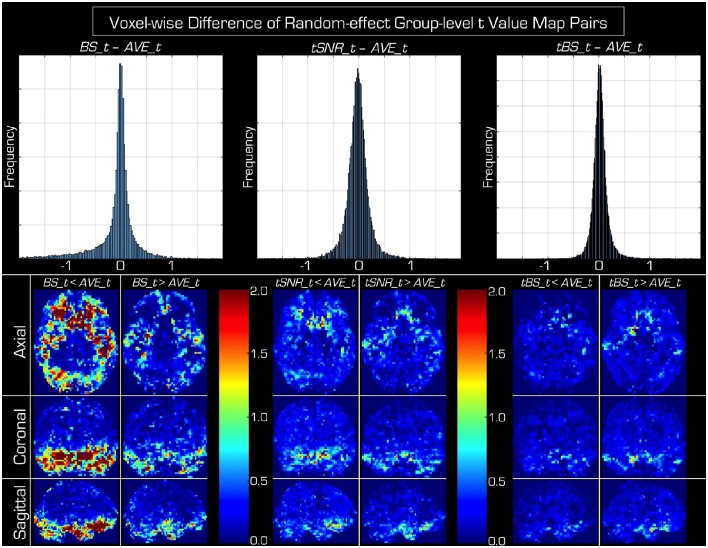
**Histograms and spatial distribution of voxels where the group-level random-effects ***t***-values differ among echo combination methods**. On the top the same data are displayed as in Figure 3, but instead of scatter plots, histograms of differences of *t*-value are given for the three advanced echo combination methods (*BS, tSNR, tBS*) relative to the *t*-values obtained from the statistical analysis using data obtained by simple averaging (i.e., *AVE_data*). In the bottom maximum intensity projections are shown in all three orthogonal orientations for the left and right tails of the corresponding histograms above. Below each histogram the left column of images marks voxels where averaging the echoes produces a higher *t*-value, while in the right column of images the particular echo combination method (BS, tSNR, tBS weighting) wins over averaging. The differences are confined to the inferior aspects of the brain and in very few voxels is there an advantage using the advanced echo combination methods.

In Figure 4 the histograms of the voxel-wise *t*-value differences of the random-effects group-level analyses within the GM segment are centered on zero (mean ± [STD], −0.11 ± [0.50] for *BS*, −0.03 ± [0.22] for *tSNR*, and 0.03 ± [0.15] for *tBS*), which is their expected value if the echo combination methods provide equivalent statistical results. However, the spreads of the histograms are not the same, showing that the spatial variance of *t*-values is dependent on the echo combination method, with *tBS* weighting showing the smallest variation (i.e., using *tBS_data* produces statistical results, which are most like that based on *AVE_data*). It is worth mentioning that the distributions of histogram differences are not symmetric. Skewness also depends on the echo combination strategy with *BS* weighting resulting in the highest asymmetry. The tails of these histograms represent the voxels where the group-level statistical *t*-values differ between the echo combination strategies. The anatomical pattern of the histograms tails is presented in the bottom of Figure 4 as maximum intensity plots. Differences between the echo combination methods are only apparent in the inferior part of the brain but the spatial arrangement of these differences precludes any possible recommendation in the optimal echo combination method.

**Figure 5:**
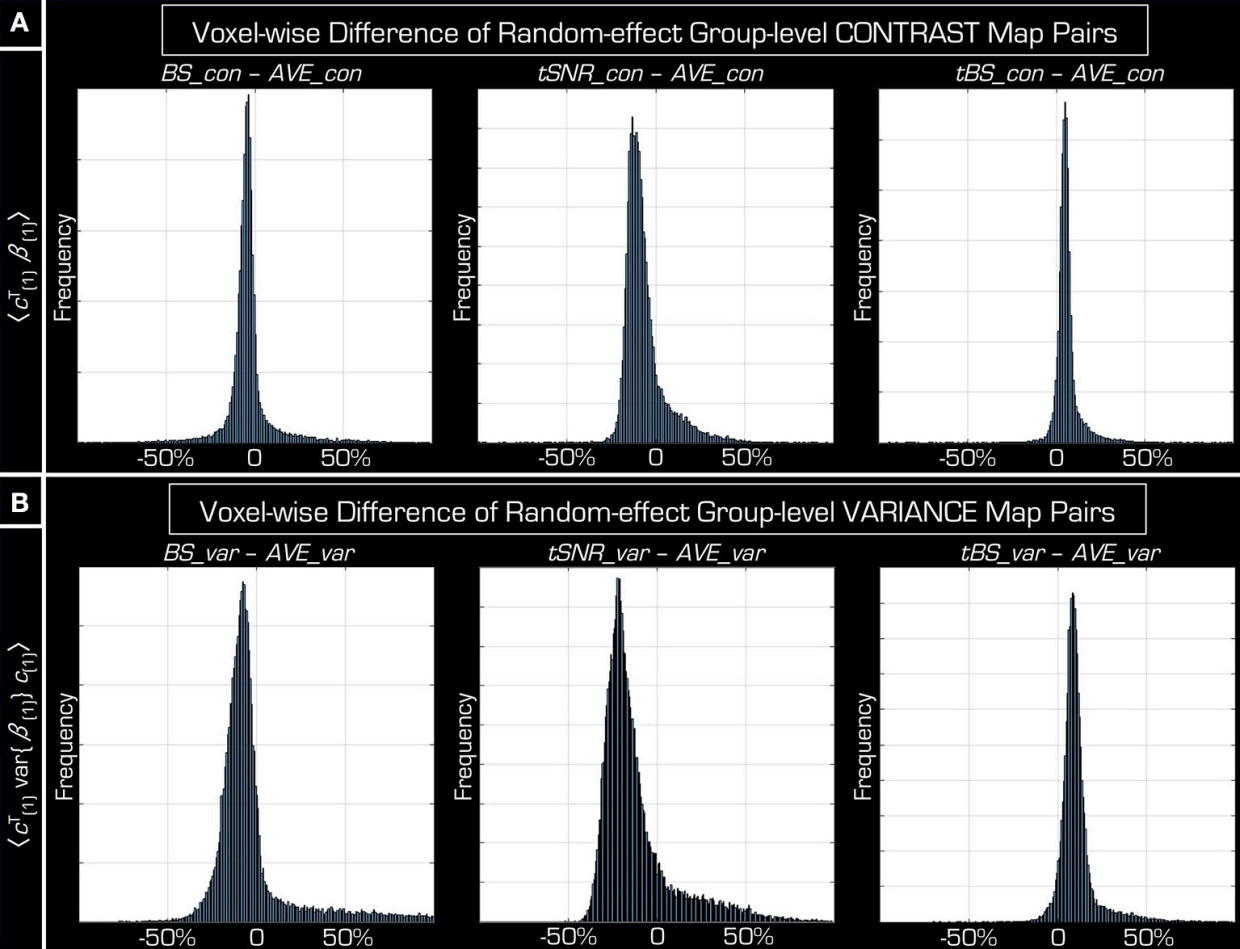
**Investigating the distribution of the components of voxel-wise ***t***-value**. Both the effect size **(A)** and the parameter variance **(B)** differ between the results obtained by simple averaging the two echoes (*AVE*) and the other three echo combination methods (*BS, tSNR, tBS*). Although, the average effect size can be clearly modulated by the different echo combination methods, variance is modulated similarly—leading to an unaffected *t*-value on average (see Figures 3, 4). *AVE_con* and *AVE_var* represent respectively the effect size and variance of the group-level random-effects statistical results performed on *AVE_data*. Similarly, BS_con, tSNR_con, tBS_con for effect size and BS_var, tSNR_var, tBS_var for variance when using data from the other three echo combination methods (BS_data, tSNR_data, tBS_data).

The group-level *t*-values are defined by both the mean contrast (i.e., the mean effect size across subjects) and the parameter variance. Further scrutiny into the *t*-value histograms from above indicates that, unlike the *t*-values themselves, the contrast is actually dependent on the method used for echo combination (mean ± [STD], −0.03 ± [0.15] for *BS*, −0.06 ± [0.12] for *tSNR*, and 0.06 ± [0.07] for *tBS*). Figure 5A shows that *tBS* weighting results in increased contrast while *tSNR* weighting and *BS* weighting have decreased mean contrast compared to echo averaging.

The reason for comparable *t*-values across the different echo combination methods can be concluded from the total variance differences in histograms (Figure 5B) (mean ± [STD], −0.03 ± [0.22] for *BS*, −0.12 ± [0.20] for *tSNR*, and 0.11 ± [0.11] for *tBS*). Based on these it can be argued that the increased spatial variance of *t*-values of *BS* weighting strategy originates from its parameter variance, as its histogram shows an asymmetric “tail” in positive direction, according to the similar “tail” on *t*-value differences histogram in negative direction in Figure 4.

**Figure 6:**
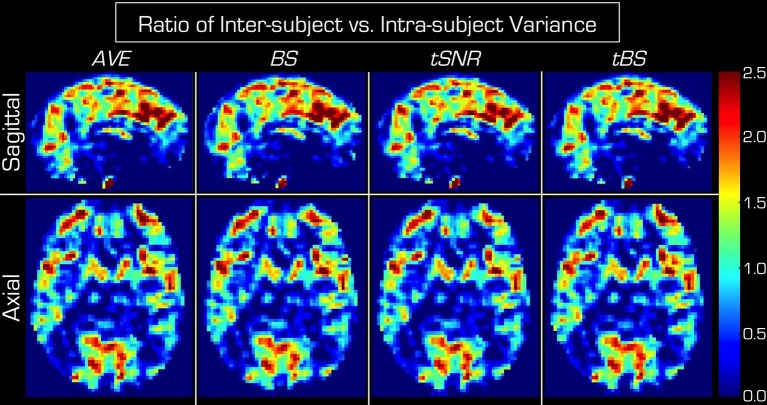
**Ratio of inter-subject vs. intra-subject variance components for each of the four echo combination methods**. In each case the inter-subject variance outweighs the intra-subject variance in large connected regions of GM. The spatial pattern is very similar across the four echo combination methods. *AVE, BS, tSNR* and *tBS* indicate the four echo combination methods. The top row displays mid-sagittal slices while the bottom row shows axial slices through the basal ganglia.

The ratio of inter-subject vs. intra-subject variance components in Figure 6 is spatially heterogeneous. In many large connected areas across the brain the intra-subject variance component is outweighed by inter-subject variance. In some smaller connected areas intra-subject variance is the dominant variance component.

**Figure 7:**
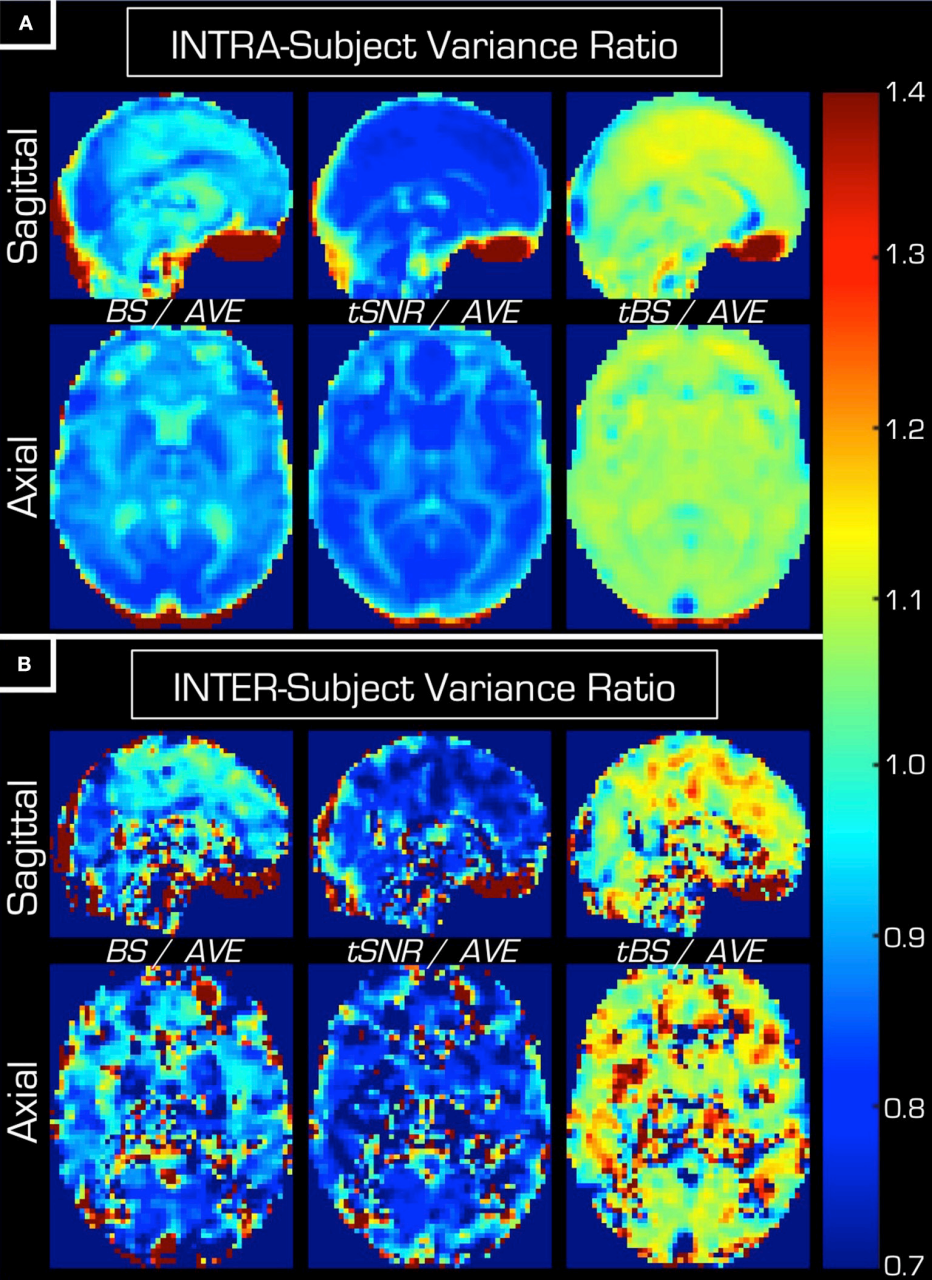
**Relating the intra-subject (A)** and inter-subject **(B)** variance components of the three advanced echo combination methods to that of the simple average. *AVE, BS, tSNR*, and *tBS* indicate the four echo combination methods. In general, using *BS* or *tSNR* weighting results in slightly smaller inter-subject and inter-subject variance components than that of the *AVE* weighting, while *tBS* weighting results in an increase in both variance components.

The actual value of both the intra-subject and the inter-subject variance components depends on the echo combination methods. In Figure 7 the variance components of data derived from the simple echo averaging (i.e., *AVE_data*) are related to those derived from the other three echo combination methods (i.e., *BS_data, tSNR_data, tBS_data*) where the intra-subject (Figure 7A) and inter-subject (Figure 7B) variance components vary similarly for a given echo combination method. Furthermore, these results from the group-level random-effect analyses of the task-based data corroborate that found in the resting-state data (Figure 1) in that an increase in inter-subject variance always follows the seemingly beneficial advanced echo combination method.

**Figure 8:**
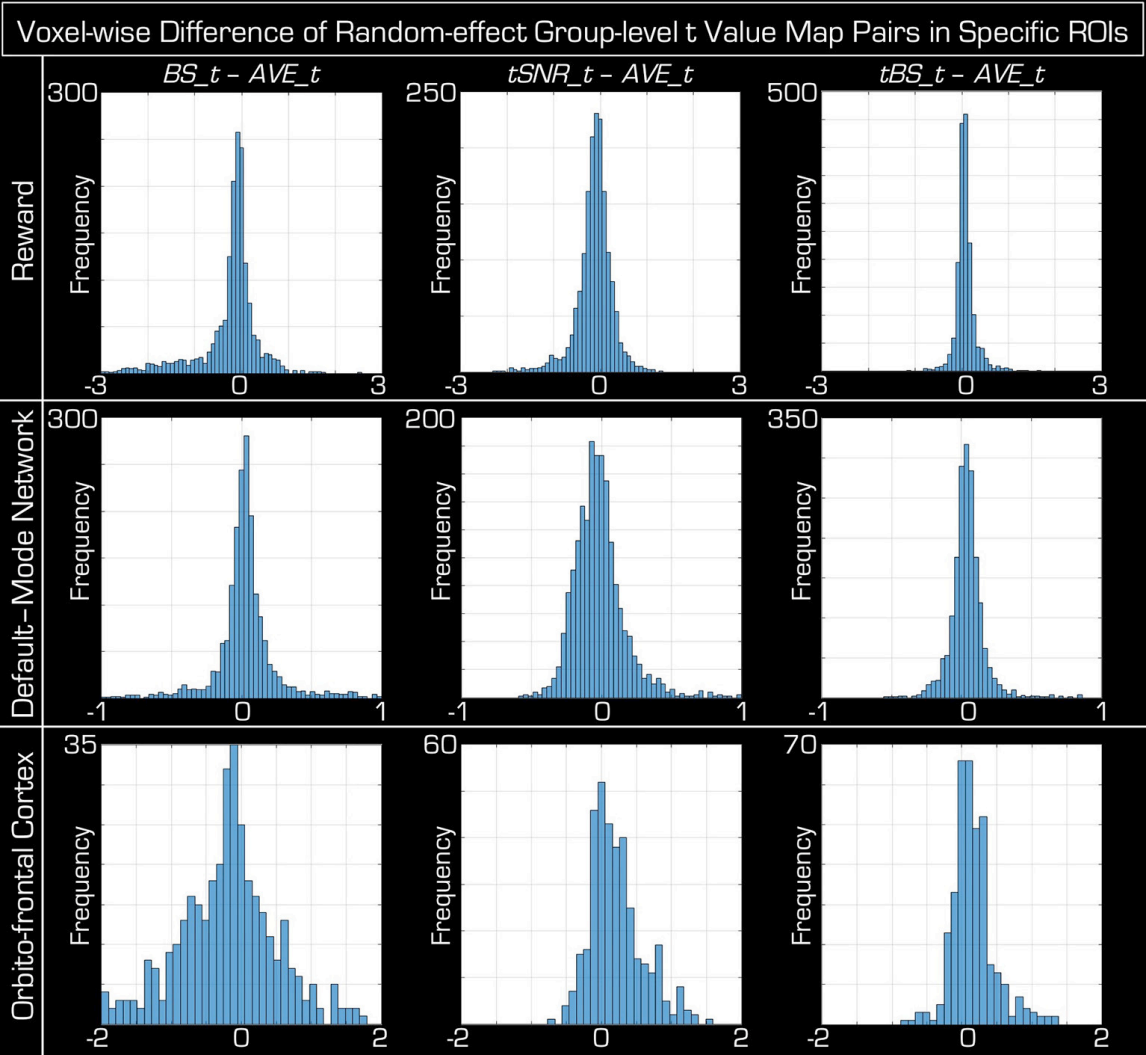
**Histograms of the voxel-wise difference in the group-level random-effects ***t***-values among echo combination methods within specific ROIs**. Across the columns, each of the three advanced echo combination methods (*BS, tSNR, tBS*) is compared against that of averaging the data. The three ROIs correspond to a Reward task (top row), the resting-state default-mode network (middle row) and the reward task activations within the orbitofrontal cortex (bottom row). Similar to the case when the entire GM segment was considered (Figure 4), the histograms are centered closed to zero and well within 1 standard deviation in each case. The limits on the horizontal axis are identical in each column but each row (i.e., each ROI) has an appropriate and unique limit.

Figure 8 displays the histograms of the voxel-wise *t*-value differences of the random-effects group-level analyses within the three ROIs (reward activation areas, default mode network, and orbitofrontal cortex). All of them are centered near zero with their mean inside the [–STD, +STD] interval, which is the result expected if the combination methods are equivalent at group level (Table 2). Similarly to the results when considering the entire GM segment (Figures 3, 4), the variance of *t*-values is dependent on the echo combination method, with *tBS* weighting showing the smallest variation, and *BS* weighting showing the largest variation in all ROIs.

**Table 2:** **The mean and standard deviation of the histogram of ***t***-value differences when the advanced echo combination methods are compared to that of the ***AVE*****.

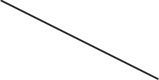	**BS**	**tSNR**	**tBS**
Reward	−0.36 ± [0.88]	0.12 ± [0.38]	0.08 ± [0.20]
DMN	0.01 ± [0.31]	−0.02 ± [0.19]	0.04 ± [0.12]
OFC	−0.24 ± [0.78]	0.21 ± [0.37]	0.16 ± [0.31]

*Numerical values are given as mean ± [standard deviation], DMN, default-mode network; OFC, orbitofrontal cortex*.

The group-level paired *t*-tests between the advanced echo combinations and the echo averaging also show that there is nearly no difference between combination methods: after the FWE correction (*p* = 0.05) there are less than 30 significant voxels scattered around all the brain, in all combination pairs (advanced vs. AVE), for both positive and negative t-contrasts.

## Discussion

We have investigated whether there is an optimal way of combining dual-echo fMRI data for random-effects group-level analysis. Surprisingly, we found that the simple averaging of the echoes (*AVE*) performs at least as well as any of the other more advanced methods. Although, the proposal for these advanced echo combination methods seems well founded theoretically and *BS* weighting and *tBS* weighting even result in an improved BOLD sensitivity (Deichmann et al., 2002; Poser et al., 2006), we found that any benefit in BOLD sensitivity on the individual level is eradicated by inter-subject variance in group-level analyses. Without significant reduction of the inter-subject variance component the slight benefits of the advanced echo combination methods will not carry over to the group level and thus the simple averaging of data from the different echoes will be sufficient.

It is important to point out that the aim of this paper does not include investigating whether multi-echo acquisitions do better or worse than single-echo variants. Although, in a very recent effort investigators reported a benefit of multi-echo acquisitions only in the orbitofrontal cortex (Kirilina et al., 2016), the aim of the present study was to consider solely multi-echo fMRI data and identify an optimal echo combination method if one exists. However, the results indicate that advanced echo combination methods do not provide a clear significant benefit over simply averaging the images that were collected with different echoes. Although the color plot at the bottom of Figure 2 indicates that in about 38% of the voxels *tBS* is the optimal echo combination method care must be taken because in 62% of the voxels it is not the best method. Furthermore, even in the orbitofrontal cortex, where multi-echo acquisition methods are superior to that of single echo methods (Poser et al., 2006; Kirilina et al., 2016) there are connected regions of voxels where one of the other three echo averaging methods provides the highest *t*-value.

Even in voxels where a robust BOLD response was detected, the advanced echo combinations failed to provide a benefit. It should be noted that elicitation of ventro-striatal brain activity by reward is among the most robust and documented fMRI findings in neuroscience (Wang et al., 2016). With such a large BOLD response one would expect more sensitivity in detecting differences between the different echo combination methods if such differences existed.

Task-based fMRI data were put through a standard preprocessing pipeline that included spatial smoothing of the images before commencing the statistical analysis, while resting-state data were left unsmoothed. This distinction is due to the fact that the resting-state data were only used for investigating the effects of echo combination methods on the BOLD sensitivity (Deichmann et al., 2002; Poser et al., 2006) (i.e., *type_ptBS* in Eq. [5]) and to calculate the *tSNR* for Echo combinations #3 and #4—but no other statistical analyses were performed.

We noted that the spread around the identity line is widest in Figure 3 when using *BS_data* for the statistical analysis. Interpretation of this finding is difficult. One may venture to speculate that the BOLD sensitivity weighting provides the least reliable *t*-values upon statistical analysis. However, there is no indication that *AVE_data* should be taken as gold standard. Here we chose to compare all other echo combination methods against the simple averaging of echoes simply because we wanted to investigate the relative advantages of more involved echo combination methods. As such, we do not mean to advocate that echo averaging is the best method. From the results of these investigations it seems that any of the echo combination methods would do just as well if for other reasons a method other than the simple average were desirable.

There are numerous sources of inter-subject variance—e.g., scanner instability, quality of image normalization processes, subject motion, respiration-induced magnetic field variations, pulsatile motion of brain due to cardiac action as well as differences in subject-dependent EPI distortion, vascular arborization in GM and cortical folding patters, to name a few. The variance component arriving from most of these sources can be reduced in principle. For example, using magnetic field probes (De Zanche et al., 2008) the effects of scanner instability or breathing on the quality of EPI images can be reduced (Kasper et al., 2014). Other possibilities are prospective motion correction, that has been shown to provide a benefit for collecting fMRI time-series data (Maclaren et al., 2013; Haeberlin et al., 2014; Todd et al., 2015), or improved shimming, that would reduce individualized susceptibility-induced distortions and drop-out artifacts. However, we may need to concede that some of the variance components, such as pulsatile brain motion or variability in brain tissue vascularization, remain beyond the experimenters' control. We stress that some sources of variance are of particular interest to researchers. Indeed, inter-individual variations in bold response may carry important information regarding variations in the underlying cognitive processes. However, within the scope of this manuscript we did not disentangle these noise components from the aforementioned variance. The current study included healthy adult volunteers and used state-of-the-art acquisition and image processing methods. In particular, we did not find excessive movement artifacts, nor did we use prospective motion correction or magnetic field monitoring methods. As such, the conclusions are expected to represent well the published fMRI literature.

Note also that in Echo combination #2, (BS), the weights are calculated separately for each time point based on the voxel signal at that time point. This can lead to an unwanted amplification of the variance in the final, combined dataset because the variance from each signal component will propagate as the square. Perhaps it is for this reason that this weighting method provides the lowest *t*-values in the group-level analysis.

It must be mentioned that apart from simply combining the echoes, as in this paper, multi-echo fMRI data can be used in additional ways to improve the sensitivity of the measurement to effects of interest. One example is employing independent component analysis to rid the data from unwanted slow drifts while maintaining slow BOLD activity patterns (Evans et al., 2015).

It is known that the effect of T2^*^ relaxation during the EPI readout period leads to blurring of the images in the phase-encoding direction because the subsequent lines of data are collected with monotonically decreasing signal intensity, as governed by T2^*^ relaxation. This effect is common for all multi-echo acquisition methods using EPI for data collection. Nonetheless, we mention it here for completeness because it is important that the weights are calculated from anisotropically blurred voxels.

One limitation of this study may be the number of echoes acquired. Multi-echo data usually includes more than two echoes and sometimes as many as six (Poser et al., 2006). It may limit the generalization of the results. For example, we could not use the T2^*^ weighting method. Nonetheless, the fact that inter-subject variance outweighs the benefits of advanced echo combinations is likely to remain the main conclusion for T2^*^ weighted echo combinations as well. Secondly, we collected a relatively short resting-state data set (~5 min). It has been shown that the reliability of functional connectivity measures improves significantly with longer acquisition (Birn et al., 2013). Because we only used the resting-state data to calculate weights for the echo combination, our results are unlikely to be negatively affected by the shorter acquisition. Finally, a notable advantage of multi-echo data, which we could not take advantage of, is the possibility of de-noising the time series which can lead to reduced inter-subject variance (Lombardo et al., 2016). However, there are settings in which collecting more than two echoes is not possible and hence dual-echo acquisition is not unprecedented (Glover and Law, 2001; Schwarzbauer et al., 2010). In those cases our results are clearly relevant. In particular, we chose to use two echoes for several practical reasons. First, we limited the SENSE acceleration factor at the modest value of 2 S, we required full brain coverage. Finally, for the task-based experiment a reasonably short repetition time was needed. With these considerations the protocol had reasonable acquisition parameters with the extra benefit of having additional data with short TE from which signal could be recovered in areas that were otherwise contaminated by drop-out artifacts.

In conclusion, we could not identify clear benefits that would make the advanced echo combination methods preferable for group-level random-effects statistical analyses, because the inter-subject variance component washes out any benefit that more intricate echo combination methods may provide on an individual level. It is important to point out that we chose the simplest echo averaging method as a reference only for convenience and would not like to advocate it in all cases. On the one hand, simple echo combination methods remove the need for collecting additional resting-state data and may be preferable in cases when time is of essence. On the other hand, if the resting-state fMRI data is available and/or subject-level analyses are planned the *BS* or *tBS* echo averaging method will slightly increase the sensitivity of the experiment.

## Author contributions

CH, AK, and ZN designed the study. CH collected the data. All authors contributed to data analysis pipeline. ZN drafted the manuscript. The submitted version is the combined effort of all authors.

### Conflict of interest statement

The authors declare that the research was conducted in the absence of any commercial or financial relationships that could be construed as a potential conflict of interest.

## Abbreviations

AVE: echo averaging
BS: BOLD sensitivity (weighting)
GM: gray matter tissue segment of the brain
tSNR: temporal signal to noise ratio (weighting)
tBS: temporal BOLD sensitivity (weighting)
STD: standard deviation
ptBS: pseudo temporal BOLD sensitivity
TE: echo time.

## References

[B1] Ashburner J. Friston K. J. (2005). Unified segmentation. Neuroimage 26, 839–851. 10.1016/j.neuroimage.2005.02.018 15955494

[B2] Birn R. M. Molloy E. K. Patriat R. Parker T. Meier T. B. Kirk G. R.. (2013). The effect of scan length on the reliability of resting-state fMRI connectivity estimates. Neuroimage 83, 550–558. 10.1016/j.neuroimage.2013.05.099 23747458 PMC4104183

[B3] Deichmann R. Gottfried J. A. Hutton C. Turner R. (2003). Optimized EPI for fMRI studies of the orbitofrontal cortex. Neuroimage 19, 430–441. 10.1016/S1053-8119(03)00073-9 12814592

[B4] Deichmann R. Josephs O. Hutton C. Corfield D. R. Turner R. (2002). Compensation of susceptibility-induced BOLD sensitivity losses in echo-planar fMRI imaging. Neuroimage 15, 120–135. 10.1006/nimg.2001.0985 11771980

[B5] De Zanche N. Barmet C. Nordmeyer-Massner J. A. Pruessmann K. P. (2008). NMR probes for measuring magnetic fields and field dynamics in MR systems. Magn. Reson. Med. 60, 176–186. 10.1002/mrm.21624 18581363

[B6] Evans J. W. Kundu P. Horovitz S. G. Bandettini P. A. (2015). Separating slow BOLD from non-BOLD baseline drifts using multi-echo fMRI. Neuroimage 105, 189–197. 10.1016/j.neuroimage.2014.10.051 25449746 PMC4262662

[B7] Fischer H. Ladebeck R. (1998). Echo-planar imaging image artifacts, in Echo-Planar Imaging: Theory, Technique and Application, eds Schmitt F. Stehling M. K. Turner R. (Heidelberg: Springer), 179–200.

[B8] Friston K. J. Ashburner J. T. Kiebel S. J. Nichols T. E. Penny W. D. (2007). Statistical Parametric Mapping: The Analysis of Functional Brain Images. London: Elsevier.

[B9] Glover G. H. Law C. S. (2001). Spiral-in/out BOLD fMRI for increased SNR and reduced susceptibility artifacts. Magn. Reson. Med. 46, 515–522. 10.1002/mrm.1222 11550244

[B10] Glover G. H. Li T. Q. Ress D. (2000). Image-based method for retrospective correction of physiological motion effects in fMRI: RETROICOR. Magn. Reson. Med. 44, 162–167. 10.1002/1522-2594(200007)44:1<162::AID-MRM23>3.0.CO;2-E 10893535

[B11] Haeberlin M. Kasper L. Barmet C. Brunner D. O. Dietrich B. E. Gross S.. (2014). Real-time motion correction using gradient tones and head-mounted NMR field probes. Magn. Reson. Med. 74, 647–660. 10.1002/mrm.25432 25219482

[B12] Hampton A. N. Bossaerts P. O'Doherty J. P. (2008). Neural correlates of mentalizing-related computations during strategic interactions in humans. Proc. Natl. Acad. Sci. U.S.A. 105, 6741–6746. 10.1073/pnas.0711099105 18427116 PMC2373314

[B13] Harvey A. K. Pattinson K. T. Brooks J. C. Mayhew S. D. Jenkinson M. Wise R. G. (2008). Brainstem functional magnetic resonance imaging: disentangling signal from physiological noise. J. Magn. Reson. Imaging 28, 1337–1344. 10.1002/jmri.21623 19025940

[B14] Kasper L. Bollmann S. Vannesjo S. J. Gross S. Haeberlin M. Dietrich B. E.. (2014). Monitoring, analysis, and correction of magnetic field fluctuations in echo planar imaging time series. Magn. Reson. Med. 74, 396–409. 10.1002/mrm.25407 25123595

[B15] Kirilina E. Lutti A. Poser B. A. Blankenburg F. Weiskopf N. (2016). The quest for the best: the impact of different EPI sequences on the sensitivity of random effect fMRI group analyses. Neuroimage 126, 49–59. 10.1016/j.neuroimage.2015.10.071 26515905 PMC4739510

[B16] Lombardo M. V. Auyeung B. Holt R. J. Waldman J. Ruigrok A. N. Mooney N. (2016). Improving effect size estimation and statistical power with multi-echo fMRI and its impact on understanding the neural systems supporting mentalizing. Neuroimage 142, 55–66. 10.1016/j.neuroimage.2016.07.022 PMC5102698 27417345

[B17] Maclaren J. Herbst M. Speck O. Zaitsev M. (2013). Prospective motion correction in brain imaging: a review. Magn. Reson. Med. 69, 621–636. 10.1002/mrm.24314 22570274

[B18] Mansfield P. (1977). Multi-planar image formation using NMR spin echoes. J. Phys. C 10, L55 10.1088/0022-3719/10/3/004

[B19] Ojemann J. G. Akbudak E. Snyder A. Z. McKinstry R. C. Raichle M. E. Conturo T. E. (1997). Anatomic localization and quantitative analysis of gradient refocused echo-planar fMRI susceptibility artifacts. Neuroimage 6, 156–167. 10.1006/nimg.1997.0289 9344820

[B20] Ordidge R. (1999). The development of echo-planar imaging (EPI): 1977–1982. MAGMA 9, 117–121. 10.1007/BF02594607 10628684

[B21] Ordidge R. J. Gorell J. M. Deniau J. C. Knight R. A. Helpern J. A. (1994). Assessment of relative brain iron concentrations using T2-weighted and T2^*^-weighted MRI at 3 Tesla. Magn. Reson. Med. 32, 335–341. 10.1002/mrm.1910320309 7984066

[B22] Poser B. A. Versluis M. J. Hoogduin J. M. Norris D. G. (2006). BOLD contrast sensitivity enhancement and artifact reduction with multiecho EPI: parallel-acquired inhomogeneity-desensitized fMRI. Magn. Reson. Med. 55, 1227–1235. 10.1002/mrm.20900 16680688

[B23] Posse S. Wiese S. Gembris D. Mathiak K. Kessler C. Grosse-Ruyken M. L.. (1999). Enhancement of BOLD-contrast sensitivity by single-shot multi-echo functional MR imaging. Magn. Reson. Med. 42, 87–97. 10.1002/(SICI)1522-2594(199907)42:1<87::AID-MRM13>3.0.CO;2-O 10398954

[B24] Pruessmann K. P. Weiger M. Scheidegger M. B. Boesiger P. (1999). SENSE: sensitivity encoding for fast MRI. Magn. Reson. Med. 42, 952–962. 10.1002/(SICI)1522-2594(199911)42:5<952::AID-MRM16>3.0.CO;2-S 10542355

[B25] Schwarzbauer C. Mildner T. Heinke W. Brett M. Deichmann R. (2010). Dual echo EPI–the method of choice for fMRI in the presence of magnetic field inhomogeneities? Neuroimage 49, 316–326. 10.1016/j.neuroimage.2009.08.032 19699805

[B26] Sladky R. Friston K. J. Tröstl J. Cunnington R. Moser E. Windischberger C. (2011). Slice-timing effects and their correction in functional MRI. Neuroimage 58, 588–594. 10.1016/j.neuroimage.2011.06.078 21757015 PMC3167249

[B27] Stehling M. K. Turner R. Mansfield P. (1991). Echo-planar imaging: magnetic resonance imaging in a fraction of a second. Science 254, 43–50. 10.1126/science.1925560 1925560

[B28] Todd N. Josephs O. Callaghan M. F. Lutti A. Weiskopf N. (2015). Prospective motion correction of 3D echo-planar imaging data for functional MRI using optical tracking. Neuroimage 113, 1–12. 10.1016/j.neuroimage.2015.03.013 25783205 PMC4441089

[B29] Turner R. Ordidge R. J. (2000). Technical challenges of functional magnetic resonance imaging. IEEE Eng. Med. Biol. Mag. 19, 42–54. 10.1109/51.870230 11016029

[B30] Wang K. S. Smith D. V. Delgado M. R. (2016). Using fMRI to study reward processing in humans: past, present, and future. J. Neurophysiol. 115, 1664–1678. 10.1152/jn.00333.2015 26740530 PMC4808130

[B31] Weiskopf N. Hutton C. Josephs O. Deichmann R. (2006). Optimal EPI parameters for reduction of susceptibility-induced BOLD sensitivity losses: a whole-brain analysis at 3 T and 1.5 T. Neuroimage 33, 493–504. 10.1016/j.neuroimage.2006.07.029 16959495

[B32] Weiskopf N. Hutton C. Josephs O. Turner R. Deichmann R. (2007). Optimized EPI for fMRI studies of the orbitofrontal cortex: compensation of susceptibility-induced gradients in the readout direction. MAGMA 20, 39–49. 10.1007/s10334-006-0067-6 17268781 PMC2798023

[B33] Yarkoni T. Poldrack R. A. Nichols T. E. Van Essen D. C. Wager T. D. (2011). Large-scale automated synthesis of human functional neuroimaging data. Nat. Methods 8, 665–670. 10.1038/nmeth.1635 21706013 PMC3146590

